# Supplementary material for: The Systems Biology Research Tool: evolvable open-source software
Source: BMC Syst Biol. 2008 Jun 29;2:55. doi: 10.1186/1752-0509-2-55 (PMC2446383; doi:10.1186/1752-0509-2-55)
Supplement: Additional file 1 — SBRT Archive. An archive of the current version of the Systems Biology Research Tool. [file 1752-0509-2-55-S1.zip › sbrt-1.4.0/doc/developers_guide/api/sbrt/kernel/math/stat/comp/Correlator.html]

Correlator


|  |  |  |
| --- | --- | --- |
| **Overview** \| **Package** \| **Class** \| **Use** \| **Tree** \| **Deprecated** \| **Index** \| **Help** | |  |
| PREV CLASS   **NEXT CLASS** | **FRAMES**    **NO FRAMES**     **All Classes** |  |
| SUMMARY: NESTED \| FIELD \| CONSTR \| METHOD | DETAIL: FIELD \| CONSTR \| METHOD |  |


---


## sbrt.kernel.math.stat.comp Interface Correlator<V,T extends CorrResult>

**Type Parameters:**: `V` - the variable type.: `T` - the correlation result type.

**All Known Implementing Classes:**: KendallTau, KendallTauA, KendallTauB, RCorrelator, RKendall, RPearson, RSpearman

---

``` public interface Correlator<V,T extends CorrResult> ```

This interface is used to represent correlation statistic computers.

**Author:**
:   This interface was written and documented by Jeremiah Wright
    while in the Wagner lab.

---

| **Method Summary** | |
| --- | --- |
| `T` | `corr(java.util.Collection<java.lang.Double> xValues, java.util.Collection<java.lang.Double> yValues)`             Computes and returns the correlation statistic of the provided x and y values. |
| `T` | `corr(java.lang.Double[] xValues, java.lang.Double[] yValues)`             Computes and returns the correlation statistic of the provided x and y values. |
| `T` | `corr(java.util.Map<? extends V,java.lang.Double> xValues, java.util.Map<? extends V,java.lang.Double> yValues)`             Computes and returns the correlation statistic of the provided x and y values. |

**Method Detail**

### corr

```
T corr(java.util.Map<? extends V,java.lang.Double> xValues,
       java.util.Map<? extends V,java.lang.Double> yValues)
```

:   Computes and returns the correlation statistic of the provided x and y
    values. The key sets of the provided maps need not be identical, but only
    the intersecting values will be considered.

    :   **Parameters:**: `xValues` - variables mapped to their values.: `yValues` - variables mapped to their values. **Returns:**: the results of computing the correlation statistic between the provided x and y values.

---


### corr

```
T corr(java.util.Collection<java.lang.Double> xValues,
       java.util.Collection<java.lang.Double> yValues)
```

:   Computes and returns the correlation statistic of the provided x and y
    values.

    :   **Parameters:**: `xValues` - an ordered collection of values.: `yValues` - an ordered collection of values. **Returns:**: the results of computing the correlation statistic between the provided x and y values.

---


### corr

```
T corr(java.lang.Double[] xValues,
       java.lang.Double[] yValues)
```

:   Computes and returns the correlation statistic of the provided x and y
    values.

    :   **Parameters:**: `xValues` - an array of values.: `yValues` - an array of values. **Returns:**: the results of computing the correlation statistic between the provided x and y values.


---


|  |  |  |
| --- | --- | --- |
| **Overview** \| **Package** \| **Class** \| **Use** \| **Tree** \| **Deprecated** \| **Index** \| **Help** | |  |
| PREV CLASS   **NEXT CLASS** | **FRAMES**    **NO FRAMES**     **All Classes** |  |
| SUMMARY: NESTED \| FIELD \| CONSTR \| METHOD | DETAIL: FIELD \| CONSTR \| METHOD |  |


---
